# A Strategy for Adenovirus Vector Targeting with a Secreted Single Chain Antibody

**DOI:** 10.1371/journal.pone.0008355

**Published:** 2009-12-21

**Authors:** Joel N. Glasgow, Galina Mikheeva, Victor Krasnykh, David T. Curiel

**Affiliations:** 1 Division of Cardiovascular Disease, Departments of Medicine, Obstetrics and Gynecology, Pathology, Surgery, and the Gene Therapy Center, University of Alabama at Birmingham, Birmingham, Alabama, United States of America; 2 Division of Human Gene Therapy, Departments of Medicine, Obstetrics and Gynecology, Pathology, Surgery, and the Gene Therapy Center, University of Alabama at Birmingham, Birmingham, Alabama, United States of America; 3 Department of Experimental Diagnostic Imaging, University of Texas M. D. Anderson Cancer Center, Houston, Texas, United States of America; University of Minnesota, United States of America

## Abstract

**Background:**

Successful gene therapy will require targeted delivery vectors capable of self-directed localization. In this regard, the use of antibodies or single chain antibody fragments (scFv) in conjunction with adenovirus (Ad) vectors remains an attractive means to achieve cell-specific targeting. However, a longstanding barrier to the development of Ad vectors with genetically incorporated scFvs has been the biosynthetic incompatibility between Ad capsid proteins and antibody-derived species. Specifically, scFv require posttranslational modifications not available to Ad capsid proteins due to their cytoplasmic routing during protein synthesis and virion assembly.

**Methodology/Principal Findings:**

We have therefore sought to develop scFv-targeted Ad vectors using a secreted scFv that undergoes the requisite posttranslational modifications and is trafficked for secretion. Formation of the scFv-targeted Ad vector is achieved via highly specific association of the Ad virion and a targeting scFv employing synthetic leucine zipper-like dimerization domains (zippers) that have been optimized for structural compatibility with the Ad capsid and for association with the secreted scFv. Our results show that zipper-containing Ad fiber molecules trimerize and incorporate into mature virions and that zippers can be genetically fused to scFv without ablating target recognition. Most importantly, we show that zipper-tagged virions and scFv provide target-specific gene transfer.

**Conclusions/Significance:**

This work describes a new approach to produce targeted Ad vectors using a secreted scFv molecule, thereby avoiding the problem of structural and biosynthetic incompatibility between Ad and a complex targeting ligand. This approach may facilitate Ad targeting using a wide variety of targeting ligands directed towards a variety of cellular receptors.

## Introduction

Successful gene therapy will require both rational vector development and exploitation of disease-specific cellular physiology to design targeted gene delivery vectors. Vectors based on human adenovirus (Ad) serotypes 2 and 5 of species C continue to show increasing promise as gene delivery vehicles due to several key attributes: Ad vectors display *in vivo* stability and excellent gene transfer efficiency to numerous dividing and non-dividing cell targets, do not integrate into the host genome, and are rarely linked to any severe disease in immunocompetent humans. Further, production parameters for clinical grade Ad vectors are well established. As of 2008, Ad vectors were employed in one-fourth of gene therapy clinical trials worldwide [1]. However, limited efficacy in clinical trials using Ad-based agents has clearly exposed the need for vector modifications designed to provide target cell-specific gene delivery and expression, thereby improving efficacy and safety.

Targeted gene delivery is ultimately predicated on the ability of the vector to discriminate between target and non-target cells via interaction with unique cell- or disease-specific surface markers. Antibodies and recombinant antibody binding domains are potentially useful agents to achieve cell-specific targeting, due to their unparalleled affinity and specificity of binding to a wide range of target cell surface markers. On this basis, the development of Ad vectors with genetically incorporated antibody-derived moieties has been a long-standing goal. Genetic capsid incorporation of several classes of attractive targeting ligands, including single-chain antibodies (scFv) and growth factors, has been severely hampered by the innate biosynthetic incompatibilities between these targeting ligands and Ad capsid proteins. Ad capsid proteins are translated and fold within the reducing environment of the cytoplasm followed by nuclear transport and virion assembly in the nucleus. In contrast, translation, post-translational modification and correct folding of scFv occur in the oxidizing environment of the endoplasmic reticulum, followed by trafficking to the cell surface via the Golgi system and subsequent secretion [2]. Thus, cytoplasmic routing of an Ad capsid-scFv fusion protein inhibits the formation of a correctly folded scFv capable of antigen recognition while also reducing Ad virion assembly and replication [3].

On this basis, we have developed a strategy to produce scFv-targeted Ad vectors that retains the native secretory biosynthetic pathway of standard available “off the shelf” scFv molecules. The unique aspect of this approach is the genetic tagging of the Ad capsid and the scFv with synthetic leucine zipper-like dimerization domains that provide high affinity, selective interaction between Ad particles and the scFv following lysis of the producer cells. In this report, we outline the construction, rescue and targeting characterization of a novel scFv targeted Ad vector, and demonstrate that this approach can provide selective and target antigen-dependent gene delivery via use of a traditional scFv molecule.

## Results

### A Strategy for Coupling a Secreted scFv to Adenovirus Using Leucine Zipper Heterodimers

**Figure 1:**
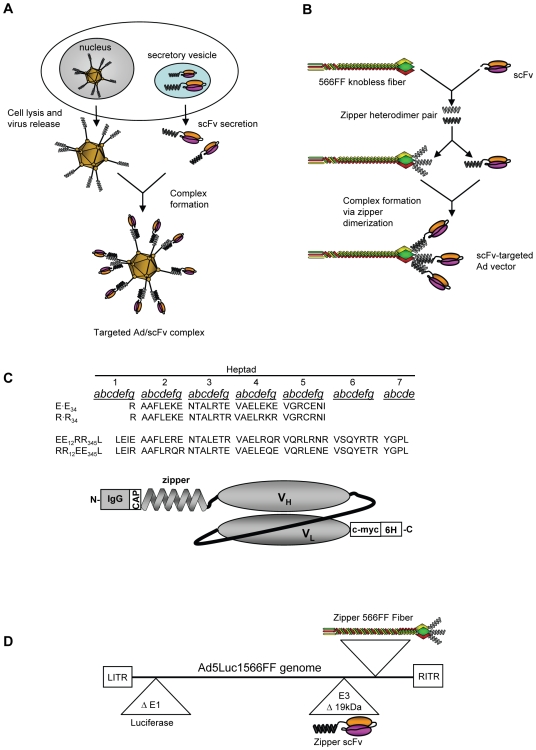
Diagrams depicting our targeting strategy, fiber and scFv configuration, zipper sequences and vector genomes. (A) Schematic of the proposed Ad vector targeting approach showing Ad-mediated scFv expression and secretion followed by cell lysis and Ad virion release and extracellular formation of a targeted Ad vector/scFv complex. (B) Configuration of heterodimeric zipper domains, wherein one zipper domain is genetically incorporated onto the C-terminus of the knobless 566FF fiber, and its counterpart is fused to the N-terminus of a recombinant scFv molecule. (C) Peptide sequences of the two heterodimeric zipper pairs used. Each of the peptides comprising the pairs E·E_34_/R·R_34_, and EE_12_RR_345_L/RR_12_EE_345_L can associate with its complimentary partner, thereby forming a stable heterodimer. The letters indicate the residue position within each heptad repeat. Also shown is a molecular map of the zipper-scFv fusion proteins showing the N-terminus, IgG-derived secretion signal peptide, a helix cap sequence, zipper sequence, a flexible linker and the scFvG28-5 followed by c-myc and 6His tags at the C-terminus. See main text for additional details. (D) Simplified genomic map of AdLuc1566FF-R/E-G28-5 and AdLuc1566FF-ER/RE-G28-5 vectors showing the CMV/firefly luciferase expression cassette in the deleted E1 region, the zipper-scFv G28-5 open reading frame in place of the 19 kDa protein locus in the E3 region, and zipper-566FF fiber molecule replacing the Ad fiber.

The ability to use a commonly available “off-the-shelf” recombinant antibody species, especially those in scFv format, for the purpose of Ad vector targeting would be highly advantageous. To this end, we have developed a system that maintains the divergent biosynthetic pathways of the antibody and adenovirus components via use of a secreted scFv molecule encoded by the vector. This design aspect requires that self-assembly of the targeted Ad vector occurs following lysis of producer cells (Fig. 1A). The ideal assembly mechanism between the scFv and the Ad virion would provide stable, selective and high-affinity association without forming aggregates. Further, the linkage function would be based on genetically incorporable peptide motifs that are small, soluble, and fully compatible with scFv structure and antigen binding, as well as with Ad capsid proteins. Based on these considerations, we chose to use a genetic approach based on engineered heterodimerizing coiled-coil protein structures derived from vitellogenin gene binding protein (VBP), a leucine zipper DNA binding protein [4]. We hypothesized that genetic tagging of the Ad virion and the scFv with a single “zipper” dimerization domain from a heterodimerizing pair would provide the stringent self-assembly properties required for the formation of scFv-targeted Ad vectors (Fig. 1B). We selected two pairs of heterodimerizing zipper domains based on size, charge and heterodimerization properties (Fig. 1C). The 29-residue E-E_34_/R-R_34_ zipper pair has been used to non-covalently crosslink GFP to proteins that are not functional as genetic fusions, and selectively forms heterodimers with a dissociation constant of K_d_ = 7×10^−9^ M and a T_m_ of 51.5°C [5], [6]. We employed a second 43-residue zipper pair, EE_12_RR_345_L/RR_12_EE_345_L, that heterodimerizes with a T_m_ of 73°C, with a corresponding dissociation constant of K_d_ = 1.3×10^−11^ M [7]. For simplicity, E-E_34_, R-R_34_, EE_12_RR_345_L and RR_12_EE_345_L zippers will also be referred to as E, R, ER and RE, respectively.

### Design of Fusion Proteins Containing Zipper Peptides and scFv

**Figure 2:**
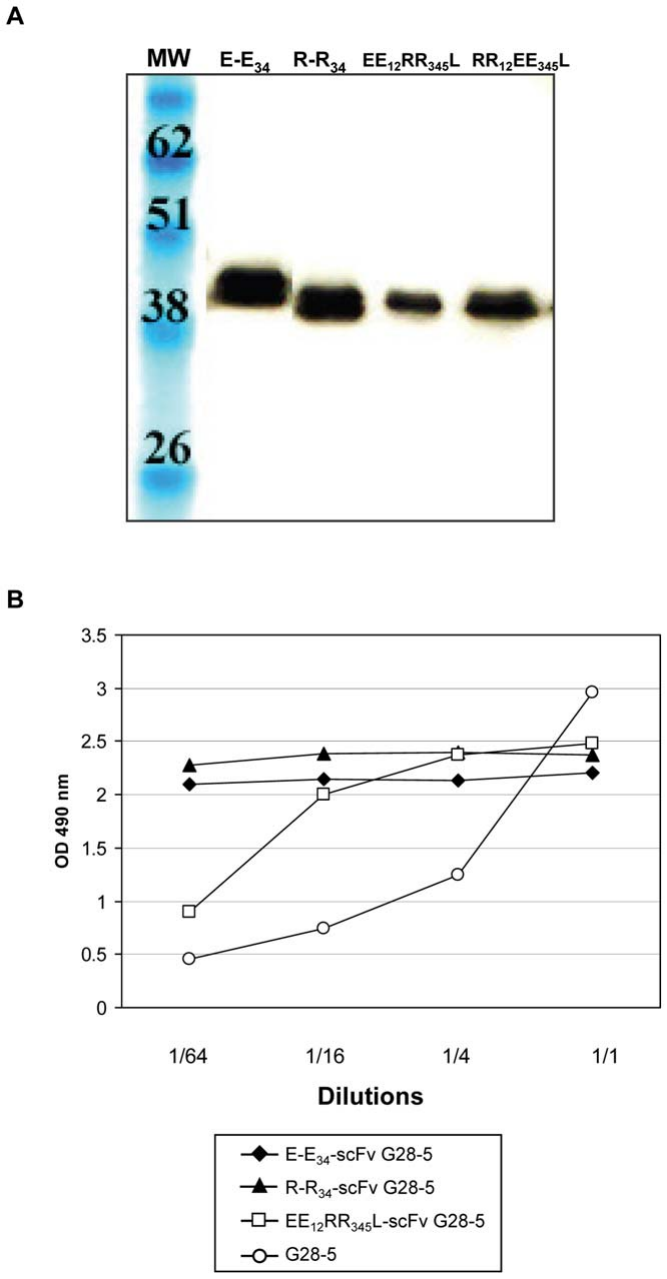
Analysis of expression and binding properties of secreted zipper-tagged scFv. (A) Western blot analysis of culture medium from A549 human lung carcinoma cells transduced with Ad5 vectors encoding each zipper-scFv G28-5 molecule. Lanes denote the zipper domain fused with each scFv. Culture medium was collected, Laemmli buffer added, and samples boiled prior to SDS-PAGE and western analysis with an anti-Penta-His antibody. Molecular mass markers indicate kilodaltons. (B) ELISA-based binding assays using metal affinity-purified zipper-G28-5 proteins. Each well contained 500 µg of CD40. Zipper-scFv G28-5 proteins were added to the wells at the dilutions indicated. The positive control parental G28-5 mAb was added at dilutions of 1/500, 1/2,500, 1/5,000 and 1/10,000 from a stock solution of 0.15 mg/ml.

Our targeting system is based on Ad vector targeting via scFv-mediated binding to a cell surface molecule. On this basis, we employed a scFv derived from the mouse monoclonal G28-5 antibody (denoted throughout as scFv G28-5) directed against human CD40 [8]. The zipper-scFv G28-5 fusion molecules were constructed to incorporate, from N- to C-terminus, a 21-residue secretion signal peptide of the kappa-chain of human immunoglobulin, a 4-residue helix cap sequence, one of the four zipper sequences, a 14-residue flexible linker and the scFvG28-5 followed by c-myc and 6His tags at the C-terminus (Fig. 1C). The four zipper-scFv G28-5 variants were incorporated into shuttle vectors, transferred into the E1 region of Ad5 genomes, and the resultant viruses were used to express each zipper-scFv G28-5 variant in A549 lung carcinoma cells. We confirmed the expression and secretion of each zipper-scFv G28-5 protein via western blot analysis of cell culture medium. As shown in Figure 2A, all four zipper-scFv proteins were detected via an anti-penta-His primary antibody in medium samples with molecular masses of approximately 39 kDa, consistent with the predicted mass of the full length proteins. No corresponding bands were present in negative control mock infected cells (data not shown). We next sought to confirm that the addition of the zipper domains to scFv G28-5 does not interfere with antigen recognition. We performed ELISA-based binding assays using metal affinity-purified zipper-G28-5 proteins and a recombinant form of the CD40 target receptor as bait. As shown in Figure 2B, scFv G28-5 fused with the E-E_34_, R-R_34_ and EE_12_RR_345_L zipper domains exhibited levels of binding comparable to the parental G28-5 mAb. These data show that zipper-tagged scFv G28-5 proteins are properly secreted and soluble when expressed via an Ad vector and, most importantly, retain native antigen recognition.

### Incorporation of Zipper Peptides in to the Ad Capsid

We next sought to determine the optimal capsid locale and genetic configuration for the display of zipper domains. We considered the key issue of the structural compatibility of zipper peptides at various capsid locales and selected the fiber as the site of zipper domain incorporation. Heterologous peptides have proven functional in the context of the Ad5 fiber C-terminus and H-I loop as well as the C-terminus of knobless artificial fibers.[9]–[13] On this basis, we incorporated each of the four zipper domains into either the C-terminus or the H-I loop of the wild type fiber protein. Also, a modified fiber protein containing extended peptide linkers within the H-I loop was used for zipper peptide display. Further, we also incorporated each zipper into the C-terminus of two knob-deleted chimeric fibers, the fiber-fibritin fiber and the 566FF fiber [11], [14], [15]. The 373-residue fiber-fibritin chimeric fiber [11] is comprised of the Ad5 fiber tail domain and two amino-terminal repeats of the Ad5 shaft domain genetically fused with eight C-terminal coiled-coil segments of the bacteriophage T4 fibritin protein. The 566FF fiber [14], [15] contains the entire Ad5 fiber shaft fused to the 12th coiled-coil segment of fibritin. A total of eighteen recombinant fiber genes were generated and then screened on the basis of protein expression, trimer formation, virion incorporation and the ability of the fiber-displayed zipper domain to heterodimerize with its corresponding zipper domain. To this end, expression plasmids encoding each zipper-containing fiber protein were transfected into HEK 293 cells and fiber expression and trimerization evaluated via western blot analysis. Of the eighteen fibers, only the fiber with the R-zipper in the H-I loop was not expressed at a level comparable to that of wild type Ad5 fiber, and was not used further (Figure S1). We observed decreased trimerization in fibers with zipper domains incorporated into the Ad5 fiber C-terminus, including R-zipper and ER-zipper containing fibers. Further, fibers with R- and ER-zippers encoded within the H-I loop exhibited decreased trimerization as judged by the lower ratio of trimeric to monomeric protein band intensity in unboiled samples, and were not used further (Figure S1).

We next sought to determine the ability of the zipper-containing fiber proteins to be incorporated into Ad virions using a fiber trans-complementation method developed by Jakobczak [16]. To do this, 293 cells were transfected with the fiber-encoding plasmids and then infected with an Ad vector which lacks the fiber gene in its genome but contains the wild type fiber in the capsid supplied by producer cells that express Ad5 fiber. Therefore, if functional, the zipper-fiber proteins produced in the cells are expected to incorporate into the capsids of the fiber-deleted virus progeny. We then analyzed virions produced by this method by western blot to determine capsid incorporation of transiently expressed zipper-fibers. Consistent with the poor trimerization we observed, fibers with zipper domains incorporated into the H-I loop were poorly incorporated into mature virions, with the exception of the fiber containing the E-zipper in the H-I loop (Figure S2). Fibers with zippers incorporated at the fiber C-terminus showed moderate to full incorporation, an interesting outcome in light of the relatively poor trimerization observed for these fibers. The remainder of the fibers showed capsid incorporation similar to that of Ad5 fiber (Figures S2 and Table 1).

**Table 1:** Summary of expression, trimerization, capsid incorporation and dimerization of zipper-modified Ad fibers.

Site of zipper incorporation	Zipper	Expression	Trimerization	Incorporation into virions	Heterodimer binding to zipper scFv	Homodimer binding to zipper scFv
Ad5 fiber	none	+++	+++	*+++*	-	-
HI-loop	E-E_34_	+++	++	+++	-	nt
	R-R_34_	+	-	**-**	nt	nt
	EE_12_RR_345_L	+++	+	**-**	nt	nt
	RR_12_EE_345_L	+++	++	**-**	nt	nt
Fiber C-terminus	E-E_34_	+	++	++	nt	nt
	R-R_34_	+	+	++	nt	nt
	EE_12_RR_345_L	++	+	++	+	-
	RR_12_EE_345_L	++	++	+++	nt	nt
Extended HI loop	E-E_34_	++	+++	+++	nt	nt
	R-R_34_	++	+++	+++	-	nt
	EE_12_RR_345_L	++	+	+++	nt	nt
	RR_12_EE_345_L	++	++	+++	-	-
C-terminus of fiber-fibritin chimera	E-E_34_	+++	++	+++	-	-
	R-R_34_	+++	+++	++	++	-
	EE_12_RR_345_L	+++	+++	nt	+++	-
	RR_12_EE_345_L	+++	+++	nt	nt	nt
C-terminus of 566FF fiber	R-R_34_	+++	+++	+++	+++	-
	EE_12_RR_345_L	+++	+++	++	++	-

“+++”, -highly positive.

“++”, -positive.

“+”, -slightly positive.

“-”, -negative result.

“nt”, -not tested.

**Figure 3:**
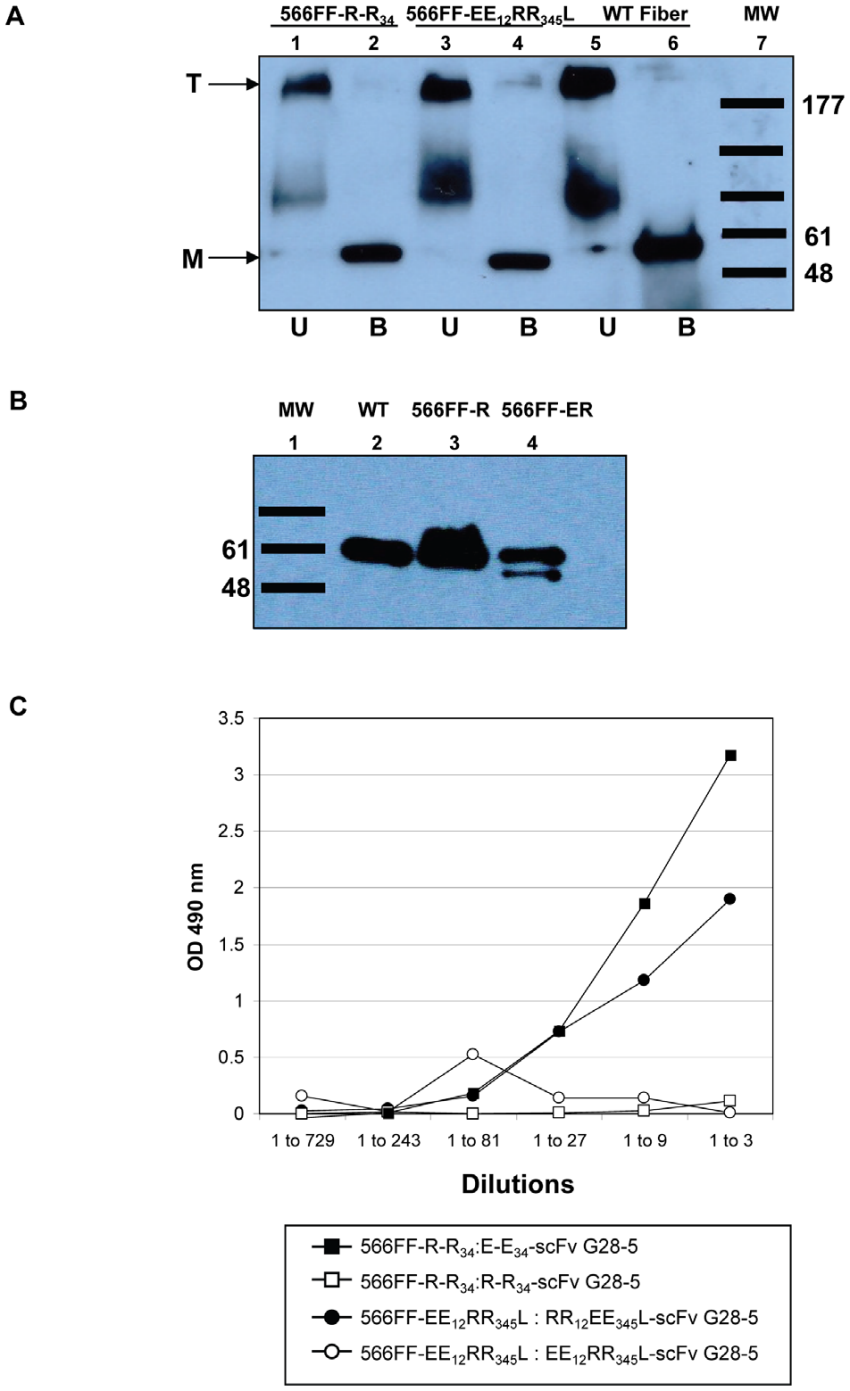
Trimerization profiles of zipper-modified 566FF fiber proteins. (A) Western blot analysis of lysates of 293T cells transfected with expression vectors expressing zipper-modified 566FF fiber proteins. Protein samples in Laemmli buffer were separated by SDS-PAGE with prior heat denaturation by boiling (B, boiled) or without boiling (U, unboiled). Following transfer of proteins to PVDF membrane, proteins were then probed with an anti-fiber monoclonal antibody that recognizes the fiber tail. Lanes 1 and 2 contains 566FF-R-R_12_ fibers, lanes 3 and 4 the 566FF-EE_12_RR_345_L fibers and lanes 5 and 6 wild type Ad5 fiber. Fiber monomers (M) and trimers (T) are indicated. Molecular mass markers indicate kilodaltons. (B) Western blot analysis of fibers from virions purified on 293 cells and containing a single genetically encoded fiber. Lane 2 contains WT Ad5 fiber, Lane 3 is 566FF-R and lane 4 is 566FF-ER. Each lane contained 5×10^9^ viral particles. (C) ELISA-based binding assay of zipper-tagged 566FF fibers with zipper-scFv G28-5. Zipper-scFv proteins expressed by Ad vectors cells were purified by immobilized metal ion affinity chromatography and adsorbed into wells of a 96-well ELISA plate. Dilutions of equal amounts of cell lysate from 293T cells transfected with plasmids encoding the 566FF-zipper proteins were added to the wells. The formation of stable complexes was demonstrated by detection of the scFv-bound 566FF fiber proteins with an anti-fiber 4D2 monoclonal antibody.

We next performed ELISA-based binding assays to determine the ability of the fiber-incorporated zipper domains to form heterodimers with the corresponding zipper-scFvG28-5 species. Purified zipper-scFv G28-5 protein was adsorbed onto ELISA plates followed by the addition of purified virions containing zipper fibers or lysates from 293 cells transfected with zipper-fiber protein expression plasmids. The formation of heterodimeric fiber/scFv complexes was demonstrated by detection of bound zipper-fiber proteins with an anti-fiber antibody or bound virions with anti-Ad anti-serum. Of note, zipper-containing fibers with poor trimerization and/or very low virion incorporation were not tested. Importantly, the results of this binding analysis indicated that maximum zipper heterodimer formation occurs with the corresponding zipper-scFv when the R-R_34_ and EE_12_RR_345_L zipper domains are located on the C-terminus of either the 566FF fiber or the fiber-fibritin knob deleted fibers (Table 1). Based on the overall performance of the 566FF fiber platform, we considered the display of the R-R_34_ and EE_12_RR_345_L zipper domains at the 566FF C-terminus to be the optimal configuration for the capsid incorporation of zipper domains. In this regard, Figure 3A shows both 566FF-R-R_34_ and 566FF-EE_12_RR_345_L fibers form trimers (lanes 1 and 3) that were converted to monomers upon heat denaturation (lanes 2 and 4), similar to wild type fiber controls. We also examined incorporation of 566FF-R-R_34_ and 566FF-EE_12_RR_345_L fibers in purified virions containing only the modified fiber. As shown in Figure 3B, both 566FF-zipper fibers were incorporated into mature virions; the 566FF-R-R_34_ fiber was incorporated fully (Figure 3B, lane 3) while the 566FF-EE_12_RR_345_L was incorporated at a reduced level. Figure 3C shows formation of heterodimeric fiber/scFv complexes with these fibers contained in cell lysates from 293 cells transfected with fiber expression vectors. Specific formation of heterodimers was observed for the 566FF-R-R_34_ and 566FF-EE_12_RR_345_L fibers and the corresponding zipper-scFv proteins (E-E_34_-scFv G28-5 and RR_12_EE_345_L-scFv G28-5, respectively). Importantly, formation of homodimeric complexes was not observed.

### Secreted scFv Provides Antigen-Specific Ad Vector Gene Transfer

**Figure 4:**
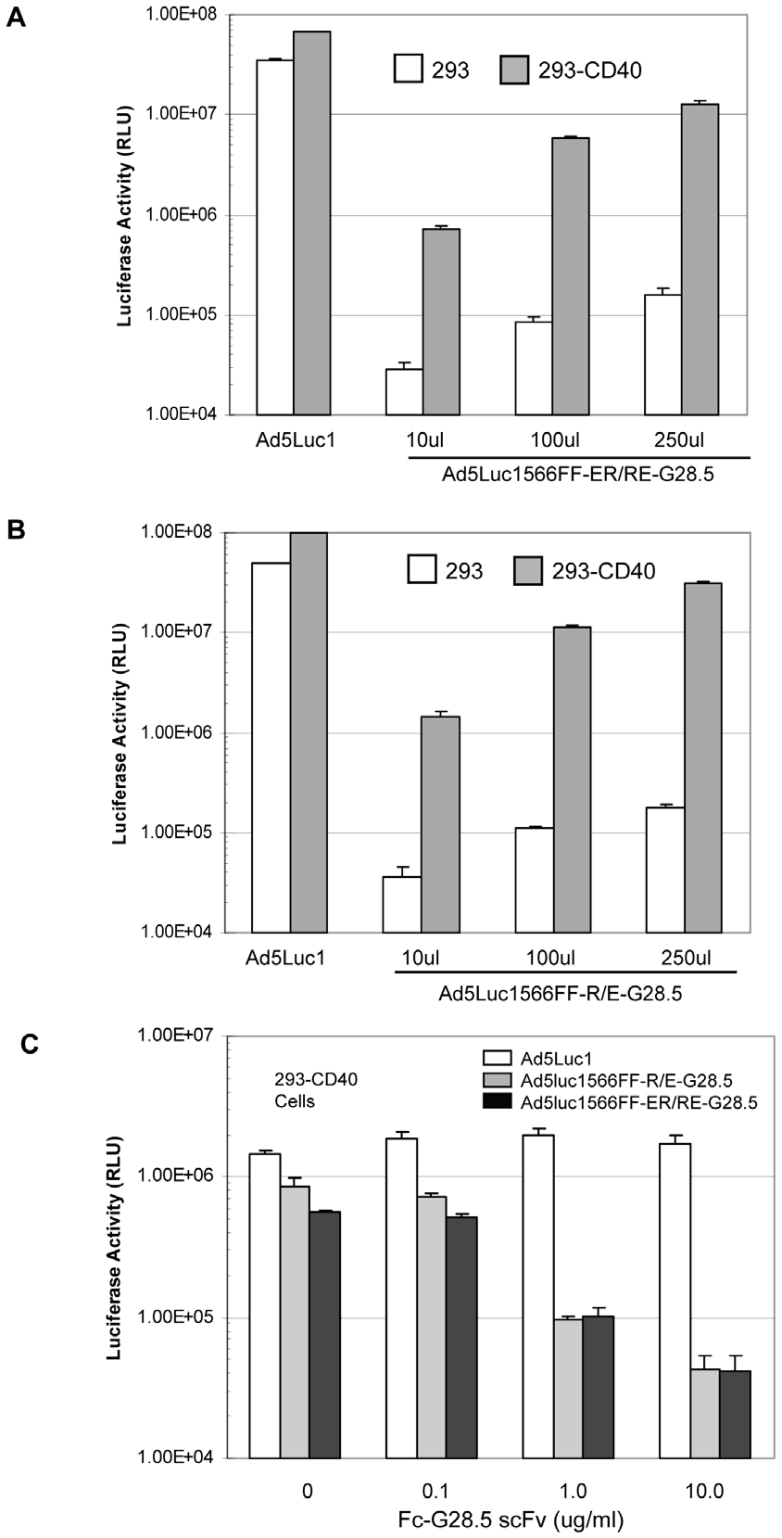
Gene transfer of scFv-targeted vectors. Each panel shows firefly luciferase activity 24 hours following transduction. Gene delivery of AdLuc1566FF-ER/RE-G28-5 vector (A) or AdLuc1566FF-R/E-G28-5 vectors (B) to CD40-negative 293 cells (white columns) or 293.CD40 cells (gray columns) is shown. Targeted vector present in culture media of 293 producer cells was added to cells cultured in 24-well plates at volumes of 10 µl, 100 µl and 250 µl per well. A control vector, Ad5Luc1 containing an isogenic reporter gene cassette, was added at 10 vp/cell. (C) Luciferase activity in 293.CD40 cells 24 hours following transduction with AdLuc1566FF-ER/RE-G28-5 and AdLuc1566FF-R/E-G28-5 targeted vectors contained in 100 µl of culture media, as above. Free Fc-scFv G28-5 was used as a competitive inhibitor of CD40 binding and was added to the wells at the concentrations indicated prior to vector addition. For all panels, each column is average of 4 replicates and error bar indicates standard deviation.

We next designed two Ad5-based vectors to evaluate the ability of our scFv-based targeting system to provide antigen-specific Ad vector gene delivery. Recombinant Ad genomes were constructed to encode a CMV promoter/firefly luciferase expression cassette in the E1 region, the 566FF-R-R_34_ or 566FF-EE_12_RR_345_L fiber and the complementary zipper-scFv G28-5 inserted into the intact E3 region in the place of the deleted 19 kDa protein (Fig. 1D). The resultant Ad vectors, designated AdLuc1566FF-R/E-G28-5 and AdLuc1566FF-ER/RE-G28-5, were rescued in fiber–expressing 293F28 cells [12]. These vectors were then propagated in 293F28 cells and purified by standard methods and the presence of both the zipper fibers and Ad5 fiber in purified virions was confirmed via western analysis (Figure S3). To generate fully targeted virions, we transduced 293 cells with purified dual-fiber AdLuc1566FF-R/E-G28-5 or AdLuc1566FF-ER/RE-G28-5 vectors, and the cells were maintained until full CPE was observed. In our system, the progeny virions released into the culture medium would contain only 566FF-zipper fibers and would form targeted complexes with secreted zipper-scFv G28-5 antibodies present in the culture medium (Fig. 1A). We next tested the ability of the AdLuc1566FF-R/E-G28-5 and AdLuc1566FF-ER/RE-G28-5 vectors to provide scFv-mediated targeted gene transfer to CD40-positive cells. We exposed CD40-negative 293 cells and the CD40-positive 293.CD40 cell line [12] to culture medium containing AdLuc1566FF-R/E-G28-5 or AdLuc1566FF-ER/RE-G28-5 vectors. As shown in Figure 4A, the non-targeted Ad5Luc1 control vector (MOI = 10 vp/cell) provided robust transgene delivery as measured by firefly luciferase activity to both 293 and 293.CD40 cell lines in a manner consistent with the similar levels of the CAR receptor in both cell lines. In contrast, gene transfer of the targeted AdLuc1566FF-ER/RE-G28-5 vector to 293.CD40 cells was increased by up to 84-fold compared to CD40-negative 293 cells. We observed a similar pattern with the AdLuc1566FF-R/E-G28-5 vector, whereby gene transfer to 293.CD40 cells was increased by up to 175-fold compared to CD40-negative 293 cells (Fig. 4B). To further confirm the capacity of the scFv-targeted vectors to deliver cell-specific gene transfer, we performed blocking experiments based on competitive inhibition using a purified form of the G28-5 scFv that is fused to an Fc domain (Fc-G28-5) [17]. As shown in Figure 4C, gene transfer to 293.CD40 cells by untargeted Ad5Luc1 control vector was not affected by the addition of free Fc-G28-5. However, gene transfer of both targeted vectors was blocked over 80% in the presence of 1.0 µg/ml free Fc-G28-5 and over 90% at 10.0 µg/ml, confirming the role of CD40 and the scFv-G28-5 in targeted gene transfer exhibited by these vectors.

## Discussion

The goal of this study was to establish a genetic Ad vector targeting platform that would allow the use of available complex peptide ligands with high target affinity and specificity, but lack the biosynthetic compatibility required for genetic fusion with Ad capsid proteins. Capsid incorporation of several classes of complex targeting ligands, including single-chain antibodies (scFv) and secreted growth factors, has been severely hampered by the innate biosynthetic incompatibilities between the ligand and Ad capsid proteins, resulting in unstable or insoluble ligands and/or reduced Ad replication [3]. With this limitation in mind, cytoplasmically stable scFv molecules have been generated via the use of highly stable antibody frameworks that can fold independently and support antigen-binding structures (complementarity determining regions or CDRs) (reviewed by M. Stocks[18]). The majority of cytoplasmically stable scFv have been directed against intracellular targets for therapeutic intervention. However, those that recognize cell surface markers could, in theory, be genetically incorporated into the Ad capsid for cell antigen-specific vector targeting. In this regard, Hedley and colleagues genetically incorporated a cytoplasmically stable scFv into an artificial knob-deleted Ad fiber and demonstrated targeted gene transfer *in vitro* to cells expressing an artificial receptor containing the scFv epitope [15]. This work established the principle that genetic fusion of a cytoplasmically stable scFv with an Ad capsid protein can allow scFv functionality, antigen recognition, targeted gene delivery and that this approach is dependent on a single cytoplasmic biosynthetic pathway. While progress has been made toward a wider use of therapeutic recombinant intrabodies through improved structural optimization and rapid screening protocols, [19]–[23] the fact remains that the repertoire of intrabodies directed against clinically relevant cell surface markers is quite limited, and has hindered the development of antibody-based Ad vector targeting.

A more common approach allowing Ad targeting with biosynthetically incompatible molecules such as scFv has been the construction of heterologous bispecific molecules that cross-link the Ad virion to alternative cell surface targets. These adapters have typically been diabodies [24], [25], fusions of a scFv and the soluble ectodomain of CAR (sCAR-scFv) [8], [26], [27] or sCAR fused to growth factors [28], and are produced separately and mixed with the Ad vector prior to use. However, in the context of conditionally replicating Ad vectors (CRAds), this approach is unsuitable since the targeting moiety is lost following the initial infection. On this basis, we and others advanced the notion of Ad targeting based on expression of secreted paracrine adapter molecules encoded in the vector genome as a means of retaining targeting capacity of conditionally replicating Ad vectors (CRAds) [29]–[32].

This study was focused on the employment of a standard recombinant single chain antibody, as members of this antibody class are widely available and capable of targeting the largest repertoire of cell-surface molecules. Rather than attempting to alter intracellular trafficking and/or biosynthesis of the targeting ligand, we employed a secreted adapter approach conceptually similar to that previously used for CRAd agents. This system relies on the native intracellular routing and secretion of the scFv with subsequent self-assembly of the targeted Ad vector occurring following lysis of producer cells during vector upscaling. This design aspect was considered critical to the applicability of this system to the widest variety of currently available “off-the-shelf” targeting ligands.

Central to the success of this approach was the use of linking moieties structurally and functionally compatible with both the Ad capsid and the targeting ligand. In this regard, we sought out linking motifs that are small, stable and soluble so as to minimize potential negative effects on targeting ligand function. Additional design requirements included association of Ad virions and targeting ligand without self-aggregation of the system components or binding to natural cellular proteins. For these reasons, protein A-, biotin/avidin- and chemical crosslinking-based approaches were not considered. With a view towards *in vivo* targeting, another critical consideration was the affinity between the linking moieties in order to minimize the potential for dissociation of the targeted Ad/scFv complex. We have previously shown that Ad vector targeting *in vitro* [26] and *in vivo* [33] using sCAR-scFv adapters is enhanced by increasing the affinity between the trimeric Ad5 fiber knob and the sCAR component by using a trimeric form of the sCAR-scFv, establishing that increased affinity between vector and ligand directly translates to improved transductional targeting. In this regard, the affinity of the Ad2 and Ad5 fibers for CAR has been determined, with dissociation constants in the range of 1-to-23 nM, depending on the methods used [34]–[36]. A similar dissociation constant (K_d_ = 3 nM) has been reported for the S11 anti-fiber knob scFv used in some diabody strategies [31], [32], [37]. On this basis, we specifically sought a linking strategy that could provide increased affinities between vector and scFv ligands not possible with sCAR-scFv or other approaches. To this end, we followed an approach based on optimized heterodimeric coiled-coil protein pairs that exhibit the aforementioned design requirements as well as heterodimer dissociation constants that extend into the picomolar range.

In this report we demonstrate the feasibility of employing heterodimer peptide zipper pairs to crosslink a genetically modified Ad vector and a secreted targeting ligand. Specifically, we have shown that two distinct zipper peptides can be genetically fused to the C-terminus of knob-deleted fibers, and that this capsid proved compatible with zipper function and vector amplification. It is possible, however, that further optimization of zipper display at the fiber capsid locale may be required to achieve the maximum utility of this approach. Equally important, we show that zipper-tagged scFv molecules are properly secreted and soluble when expressed via an Ad vector and retain native antigen recognition. Further, we demonstrate that scFv-targeted Ad vectors provide gene delivery that is dependent on target antigen recognition.

We have previously shown antibody-mediated Ad vector targeting *in vivo* via the use of heterologous adapter molecules in the form of bi-specific antibody conjugates [38], [39] or scFv-containing fusion proteins [27], [33], [40]. While effective *in vivo*, these two-component systems rely on separate production and purification of the Ad vector and the adapter molecule, followed by mixing of the two components before administration. In this regard, it is generally recognized that a single component vector would likely be more suitable for regulatory approval and clinical use. The approach we describe here is a step toward development of a targeted Ad vector wherein the vector and targeting ligand are produced simultaneously within the same culture conditions, and could therefore mimic the production schemas of single component systems. In this regard, our future work will seek to establish optimal methods by which targeted Ad vectors produced in this system will be purified. An important design aspect of our system is that the targeted vectors are ablated for CAR binding, thereby eliminating the possibility of ectopic vector localization *in vivo* to CAR-expressing tissues if the targeted complex is disrupted. As a consequence, the initial rescue and propagation of the vectors is inefficient in 293 cells. For preclinical work, this issue is circumvented via the use of 293 cells that stably express Ad5 fiber, yielding an intermediate vector species that is then upscaled using standard 293 cells. At the present time, however, it is unclear whether a technically similar approach could be used for production of clinical grade CAR-ablated vectors.

In conclusion, this Ad targeting approach provides a novel way to circumvent the problem of structural and biosynthetic incompatibility between Ad and complex targeting ligands, and could facilitate Ad targeting to a wide variety of clinically important cell populations using novel targeting ligands including recombinant antibodies and growth factors.

## Materials and Methods

### Cell Lines

The Ad5 DNA-transformed 293 and 293T human embryonic cells line were purchased from Microbix (Toronto, Ontario, Canada). The 293F28 and 293/hCD40 cell lines are derivatives of 293 cells which express either Ad5 wild-type fiber (for virus propagation) or human CD40 as previously described[12]. 293F28 cells were maintained with 600 µg/ml Zeocin (Invitrogen), and the 293/CD40 cell with 1000 ug/ml G418. Human lung adenocarcinoma A549 cells were obtained from the American Type Culture Collection (ATCC, Manassas, Va.) and maintained in DMEM with 5% (v/v) fetal calf serum. All 293 and 293-derived cell lines were propagated in a 50∶50 mixture of DMEM/F-12 media supplemented with 10% (v/v) fetal calf serum (FCS), L-glutamine (2 mM), penicillin (100 units/ml) and streptomycin (100 µg/ml) at 37°C in 5% CO_2_ in humidified conditions.

### Genetic Engineering

#### Enzymes

Restriction endonucleases and T4 DNA ligase were purchased from New England Biolabs (Beverly, MA). Polymerase chain reaction (PCR) was performed with *Pfu* DNA polymerase (Stratagene, La Jolla, CA).

#### Construction of recombinant plasmids

An Ad shuttle vector suitable for modification of the C-terminus of the fiber protein was designed by subcloning an *Age*I-*Mfe*I-fragment of the previously described pBS.F5_LL_BamHI plasmid [41] into the pKan3.1 shuttle vector. The resultant plasmid, pKan-FbLL-BamHI, contains a modified fiber gene whose 3′ end is fused with a sequence encoding a short peptide linker (G_4_S)_3_ with a terminal *Bam*HI site. The gene is flanked with DNA sequences adjacent to the wild type fiber gene in the Ad5 genome.

An Ad shuttle vector suitable for modification of the C-terminus of the fiber-fibritin protein was designed by subcloning an *Age*I-*Mfe*I-fragment of the previously constructed pXK.FF.LL plasmid [12] into the pKan3.1 shuttle vector. Plasmid pXK.FF.LL contains the gene encoding the chimeric Ad5 fiber-phage T4 fibritin protein [11] fused with a C-terminal flexible linker. The resultant pKan-FF-LL-BamHI contains a modified fiber-fibritin gene whose 3′ end is fused with the sequence encoding a short peptide linker (G_4_S)_3_ with a terminal *Bam*HI site. The gene is flanked with DNA sequences adjacent to the wild type fiber gene in the Ad5 genome. To incorporate the zipper peptides into the C-terminus of the 566FF fiber [14], we employed shuttle plasmid pKan.566FF-LL-BamHI which contains a chimeric fiber comprised of the tail and shaft domains of Ad5 fiber fused to the 12th coiled-coil segment of fibritin followed by a peptide linker (G_4_S)_3_ with a terminal *Bam*HI site. The 566FF gene is flanked with DNA sequences adjacent to the wild type fiber gene in the Ad5 genome.

Four double-stranded DNA templates corresponding to each of the four zipper peptides were assembled with pairs of oligonucleotides, which formed partial duplexes upon annealing via complementary sequences (see Table S1; underlined nucleotides are complementary). The recessed 3′-ends of the resultant duplexes were then filled in a PCR-like reaction employing *Pfu* DNA polymerase (Stratagene, La Jolla, CA) to generate blunt-ended molecules. *Bam*HI sites (underlined) and stop codons (bolded) were added to these blunt-ended templates by PCR using the following primer pairs: E-E_34_ (fwd 5′- CGGGATCCCGTGCAGCTTTCCTGGAGAA, rev 5′- CGGGATCC **TTA**GATGTTCTCACATCGTCCT); R-R_34_ (fwd 5′- CGGGATCCCGTGCAGCTTTCCTGGAGAA, rev 5′-CGGGATCC **TTA**GATGTTCCGACATCGTCCT); EE_12_RR_345_L (fwd 5′-CGGGATCCCTGGAGATCGAGGCAGCTT, rev 5′-CGGGATCC **TTA**CAGAGGTCCGTAACGAGTT); RR_12_EE_345_L (fwd 5′- CGGGATCCCTGGAGATCCGTGCAGCTT, rev 5′-CGGGATCC **TTA**CAGAGGTCCGTAACGAGTT). The generated PCR products encoding the zipper peptides were cleaved with *Bam*HI and cloned into *Bam*HI-digested pKan-LL-BamHI, pKan-FF-LL-BamHI and pKan.566FF-LL-BamHI resulting in shuttle plasmids pKan-LL-E, pKan-LL-R, pKan-LL-ER pKan-LL-RE and pKan-FF-LL-E, pKan-FF-LL-R, pKan-FF-LL-RE, pKan-FF-LL-RE, pKan.566FF-LL-E and pKan.566-LL-RE

To create mammalian expression plasmids encoding the zipper-containing fibers, *Age*I-*Mfe*I or *Age*I-*Swa*I fragments for fiber or fiber-fibritin, respectively, from the above fiber shuttle vectors were subcloned into similarly digested expression plasmid pVSII [17]. Plasmid pVSII encodes the Ad5 fiber with a unique *Mfe*I site downstream from the 3′ end of the fiber open reading frame. The resulting fiber expression plasmids were designated pVS-LL-E, pVS-LL-R pVS-LL-RE, pVS-LL-ER and pVS-FF-LL-E, pVS-FF-LL-R, pVS-FF-LL-RE, pVS-FF-LL-ER pVS-566FF-R and pVS-566FF-EN.

To generate Ad5 fibers containing zipper peptides within the H-I loop of the fiber knob domain, we constructed an expression vector, pVS.ΔHI-BaeI, which contains the Ad5 fiber gene with the H-I loop sequence partially deleted and replaced with a *Bae*I restriction site. This was done by subcloning an *Age*I-*Mfe*I fragment of the previously described Ad fiber shuttle vector pKan3.1ΔHI-BaeI [17] into *Age*I-*Mfe*I-linearized pVS. Of note, *Bae*I is a type I restriction enzyme and excises its recognition sequence leaving unique, non-complementary sticky ends in the digested vector, thereby facilitating directional cloning of inserts. Sticky ends complementary to those in *Bae*I-digested pVS.ΔHI-BaeI were added to all four zipper-encoding templates generated previously (see above) by using a sticky end PCR protocol with two primer sets (see Table S2). The resultant sticky-end PCR products encoding each zipper were denatured, annealed and ligated into *Bae*I-cut pVS.ΔHI-BaeI. This resulted in a set of four pVS.ΔHI-BaeI-derived expression vectors: pVS-HI-E, pVS-HI-R, pVS-HI-ER and pVS-HI-RE. To create corresponding fiber shuttle vectors containing zipper peptides in the H-I loop, *Age*I-*Mfe*I fragments from pVS-HI-series expression vectors were ligated into *Age*I-*Mfe*I-digested pKan3.1 shuttle vector to yield shuttle plasmids pKan-HI-E, pKan-HI-R, pKan-HI-RE and pKan-HI-ER.

To generate expression vectors containing genes for fibers with zippers incorporated into an extended H-I loop, we used previously designed fiber shuttle plasmid pHI-PB40 [13] as starting material. Plasmid pHI-PB40 encodes a modified Ad5 fiber with a genetic extension of the HI-loop with a 40aa sequence from the Ad5 penton base comprised of the 20 upstream residues and 20 downstream residues that flank the native RGD tripeptide motif, but with the RGD replaced by a *Bae*I restriction site. An *Age*I-*Mfe*I-fragment of pHI-PB40 was subcloned into *Age*I-*Mfe*I-linearized pVS, resulting in expression vector pVS-PB40_(BaeI)_. Sticky ends complementary to those in *Bae*I-digested pVS-PB40_(BaeI)_ were added to all four zipper-encoding templates generated previously by using a sticky end PCR protocol using two primer sets (see Table S2). The resultant sticky ended PCR products were denatured, annealed and cloned into *Bae*I-cleaved pVS-PB40_(BaeI)_. This resulted in a set of four pVS-PB40-derived expression vectors: pVS-PB40-E, pVS-PB40-R, pVS-PB40-ER and pVS-PB40-RE. To create corresponding fiber shuttle vectors containing zipper peptides in the extended H-I loop, *Age*I-*Mfe*I fragments from pVS-PB40-series expression vectors were ligated into *Age*I-*Mfe*I-digested pKan3.1 shuttle vector to yield shuttle plasmids pKan-PB40-E, pKan-PB40-R, pKan-PB40-RE and pKan-PB-ER.

Shuttle vectors for transfer of zipper-scFv transgenes into the Ad5 E3 region are based on plasmid pZErO-2E3-6.9 that was constructed by ligating *Hin*dIII-cut pZErO-2 (Invitrogen) to a 6.9 kb *Hin*dIII fragment from fiber-deleted genome plasmid pVL5000. This fragment contains the entire E3 region, the deleted fiber region replaced by a *Swa*I site and E4 orf6. In order to modify the E3 region in pZErO-2E3-6.9 for insertion of transgenes, a 2.7 kb *Eco*R1 fragment containing the native E3 region was ligated into *Apa*I-digested pZErO-2 to yield plasmid pZErO-2E3-2.7. Next, the 19 kD gene within pZErO-2E3-2.7 was deleted by digesting with *Xba*I and *Mfe*I. A 360 bp PCR product containing *Xba*I/*Mfe*I compatible ends and a unique *Hpa*I restriction site was ligated into *Xba*I/*Mfe*I-digested pZErO-2E3-2.7 resulting in plasmid pZErO-2E3Δ19k-HpaI. Blunt-ended PCR products encoding each of four zipper-scFv cDNAs were ligated into *Hpa*I-digested pZErO-2E3Δ19k-HpaI to yield plasmids pZErO-2E3d19k-sp-E-G28, pZErO-2E3d19k-sp-R-G28, pZErO-2E3d19k-sp-ER-G28 and pZErO-2E3d19k-sp-RE-G28. To insert transgenes into the pZErO-2E3-6.9 shuttle plasmid, *Apa*I-*Xho*I fragments from the four pZErO-2E3Δ19k-HpaI–based plasmids above were ligated into similarly digested pZErO-2E3-6.9, resulting in shuttle vectors pZErO-2E3-6.9-E-G-28, pZErO-2E3-6.9-R-G-28, pZErO-2E3-6.9-ER-G-28, and pZErO-2E3-6.9-RE-G-28.

#### Generation of recombinant adenovirus

Recombinant Ad genomes incorporating only modified fiber genes were derived by homologous DNA recombination in *Escherichia coli* BJ5183 with *Swa*I-linearized genome plasmid pVK700 and fiber shuttle vectors listed above as described previously [10]. pVK700, a derivative of pTG3602 [42], contains an Ad5 genome with full E1, E3, and fiber gene deletions and a firefly luciferase reporter gene driven by the cytomegalovirus immediate early promoter in place of the E1 region.

Recombinant Ad genomes incorporating both modified fiber and zipper-modified scFv were created by two separate homologous recombinations in *Escherichia coli* BJ5183 with *Swa*I-linearized genome plasmid pVL700ΔE3, an Ad5 genome with full E1, E3 and fiber gene deletions, but with a unique *Swa*I restriction site in place of the deleted E3 and fiber regions. Plasmid pVL700ΔE3 was created by homologous recombination between *Swa*I-cut pVK700 and plasmid pZErO-2ΔE3.SwaI, a shuttle vector containing a *Swa*I-containing short linker sequence between the genomic regions flanking the E3 region and the fiber region. Plasmid pZErO-2ΔE3.SwaI is a derivative of plasmid pZErO-2E3-6.9 and was created by *Sma*I digestion of pZErO-2E3-6.9 and subsequent ligation of a 5′-GGGAAATTTAAATTCCC-3′ duplex linker containing a *Swa*I site (underlined). To insert the zipper-scFv cDNAs into the E3 region, the first homologous recombination was performed between *Swa*I-digested pVL700ΔE3 and *Swa*I-digested shuttle vectors pZErO-2E3-6.9-E-G-28, pZErO-2E3-6.9-R-G-28, pZErO-2E3-6.9-ER-G-28, and pZErO-2E3-6.9-RE-G-28. The resulting fiber-deleted genome plasmids containing the zipper-scFv in the E3 region were produced: pVL700ΔE3-E-G28-5, pVL700ΔE3-R-G28-5, pVL700ΔE3-ER-G28-5 and pVL700ΔE3-RE-G28-5. These genomes were then digested with *Swa*I and recombined with *Eco*RI-linearized fiber shuttle vectors pVS-566FF-R and pVS-566FF-EN to generate final genomes AdLuc1566FF-R/E-G28-5 and AdLuc1566FF-ER/RE-G28-5.

Ad vectors AdLuc1566FF-R/E-G28-5 and AdLuc1566FF-ER/RE-G28-5 were generated by transfection of 293F28 cells with *Pac*I-digested Ad genome vectors as described previously [12]. 293F28 cells stably express the wild-type Ad5 fiber, so the rescued virions at this point contained both wild-type fibers and 566FF-zipper fibers. These dual-fiber viruses were purified by two rounds of equilibrium centrifugation in CsCl gradients by a standard protocol [43]. To generate virions with only 566FF-ziper fibers, we transduced 293 cells with purified dual-fiber AdLuc1566FF-R/E-G28-5 or AdLuc1566FF-ER/RE-G28-5 vectors, and the cells were maintained until full CPE was observed and virions were purified as above. Viral particle (v.p.) concentration for all Ad vectors was determined at 260 nm by the method of Maizel [44] by using a conversion factor of 1.1×10^12^ v.p./absorbance unit.

### Expression of Zipper-scFv Proteins

The coding sequence for anti-CD40 scFv G28.5 [8] was fused with the sequences encoding each of the four zipper peptides and a signal peptide of the κ–chain of human immunoglobulin using a sticky-end PCR strategy (see Figure 1). The fusion genes were then ligated between the *Pme*I and *Bam*HI sites in the Ad shuttle vector pAdenoVator-CMV5 (Q-Biogene) and homogonously recombined into a Ad5 genome pAdenoVatorΔE1/ΔE3 contained in a bacterial plasmid. The four resultant genomes were used to rescue Ad vectors of interest in 293 cells as above. The viruses generated were employed to direct the expression of the zipper-ligand proteins in human lung carcinoma A549 cells. Zipper-scFv proteins expressed by these Ad vectors were purified by immobilized metal ion affinity chromatography and then used in ELISA-based binding assays.

### Expression of Zipper-Modified Fiber Proteins

Recombinant fiber genes were assembled in various Ad shuttle vectors and then transferred to the pVSII fiber expression plasmid as described above and previously [17]. Each transfection mixture consisted of 4 ug of endotoxin-free fiber-encoding pVSII expression plasmid in HEPES buffer containing 20 ul of DOTAP (Roche) transfection reagent in a total volume of 200 ul. This mixture was then diluted into 2 ml of OPTI-MEM media and applied directly to 293T cells. Cells were maintained under standard condition for 4 hours after which 3 ml complete media was added. At 48 hours post-transfection medium was removed and cells were collected in 200 ul Cell Culture Lysis Buffer (Promega) and cell debris was removed via centrifugation. Lysates were used for enzyme-linked immunosorbent assay (ELISA) and for western analysis.

### Western Blot Analysis

Protein samples were diluted into Laemmli buffer and incubated at room temperature (unboiled samples) or 95°C for 5 minutes (boiled samples) and loaded onto a 7.5% or 4–20% gradient SDS-polyacrylamide gel (Bio-Rad, Hercules, CA). Following electrophoretic protein separation, proteins were electroblotted onto a PVDF membrane. For detection of recombinant fibers, the 4D2 primary monoclonal antibody (recognizing the Ad5 fiber tail domain) was diluted 1/3000 (Lab Vision, Freemont CA). Zipper-scFv G28-5 proteins were detected via an anti-Penta-his primary antibody (Qiagen). Immunoblots were developed by addition of a secondary horseradish peroxidase-conjugated anti-mouse or anti-rabbit immunoglobulin antibodies at a 1/3000 dilution (Dako Corporation, Carpentaria, CA), followed by incubation with 3-3′-diaminobenzene peroxidase substrate, DAB (Sigma Chemical Company, St. Louis, MO).

### ELISA

The wells of 96-well Nunc Immuno-Plates (Fisher Scientific, Pittsburgh, Pa.) were coated overnight at 4°C with the target protein diluted in 50 mM carbonate buffer (pH 8.6) in a total volume of 100 µl. Target proteins were zipper-scFv G28-5 proteins expressed in A549 cell and purified from culture media or recombinant human CD40/Fc (R&D Systems, Minneapolis, MN). The unsaturated surface of the wells was then blocked for 1 h at room temperature by the addition of 200 µl of blocking buffer (Tris-buffered saline [TBS] with 0.05% Tween 20 and 0.5% casein) to each well. The blocking buffer was replaced with 100 µl of zipper-scFv G28-5 protein or 293T cell lysates containing zipper-modified fiber proteins diluted in binding buffer (TBS with 0.05% Tween 20 and 0.05% casein). Plates were incubated at room temperature for 1 h and then were washed four times with washing buffer (TBS with 0.05% Tween 20). Bound fiber proteins or Ad particles were detected by incubation for 1 h at room temperature with anti-fiber 4D2 MAb or anti-Ad2 polyclonal antibodies, respectively. The wells were washed four times with washing buffer; either goat anti-mouse immunoglobulin G or goat anti-rabbit immunoglobulin antibodies conjugated with horseradish peroxidase (Dako) were then added, and incubation was continued for 1 h. The color was developed with a Sigma FAST *o*-phenylenediamine dihydrochloride tablet kit as recommended by the manufacturer. The color intensity was measured at 490 nm with an EL800 plate reader (Bio-Tek Instruments, Winooski, Vt.).

### Gene Transfer Assays

For gene transfer experiments 293 and 293-CD40 cells were plated in wells of 24-well plates and were transduced for 1 hour at 37°C with Ad5Luc1 at an MOI of 10 vp/cell or with various volumes (10–250 µl) of culture media containing scFv-targeted Ad vectors AdLuc1566FF-R/E-G28-5 and AdLuc1566FF-ER/RE-G28-5 in a total of 300 µl of infection media containing 2% fetal calf serum. Following transduction, cells were rinsed in infection media, and were maintained at 37°C in an atmosphere of 5% CO_2_. Cells were harvested 24 hours post-transduction and gene transfer was determined using a luciferase activity assay system (Promega, Madison, WI) according to the manufacturer's instructions. For experiments containing blocking agents, recombinant Fc-G28.5 scFv was incubated at 0.1, 1.0, and 10 µg/ml final concentration with the cells at 37°C in transduction media 15 minutes prior to the addition of the virus. Following the transduction, cells were rinsed with transduction media to remove unbound virus and blocking agent, and were maintained at 37°C in an atmosphere of 5% CO_2_.

## Supporting Information

Figure S1 Western blot analysis of expression and trimerization of zipper-modified fiber proteins. The lysates of 293 cells transfected with plasmid vectors expressing zipper-modified proteins were separated by SDS-PAGE with or without prior denaturation by boiling. The proteins were then probed with anti-fiber antibody. Vectors used are shown at the top. E, R, ER, and RE indicate the peptide zippers incorporated into the proteins. WT, wild type Ad5 fiber, B and UB stand for boiled and unboiled samples, respectively.
(0.42 MB TIF)

Figure S2 Incorporation of zipper-modified fiber proteins into Ad virions. 293 cells were transfected with fiber-encoding plasmids and then infected with an Ad vector which lacks the fiber gene in its genome but contains the wild type fiber in the capsid supplied by 293F28 producer cells. Purified Ad virions were analyzed by western blot using anti-Ad5 fiber tail antibody. The blot shows heat-denatured monomeric fibers only. Localization of zipper peptides within the fibers or fiber-fibritin (FibF) chimeras is shown at the top. wt, wild-type Ad5 fiber.
(0.45 MB TIF)

Figure S3 Incorporation of dual fibers into Ad virions. Purified Ad virions were separated on a 7.5% SDS-PAGE gel and then analyzed by western blot using anti-Ad5 fiber tail antibody. The blot shows heat-denatured monomeric fibers only. Lane 2 contains purified AdLuc1566FF-R/E-G28-5 virions that contain both WT and 566FF-R fibers after propagation in 293F28 cells. Lane 3 contains purified Ad5 virions propagated in 293 cells. Lane 4 contains lysate from 293F28 cells infected with AdLuc1566FF-R/E-G28-5 showing the presence of both WT and 566FF-R fibers in the cells. Lanes 2 and 3 contained 1x10e10 viral particles. Due to the size similarity between the WT Ad5 fiber and 566FF-ER, these fibers migrate as a single band and are not shown.
(0.29 MB TIF)

Table S1 Oligonucleotides used for assembling sequences encoding peptide zippers. When partially annealed, the underlined complementary nucleotides form a duplex within each zipper sequence. The recessed 3′-ends of the resultant duplexes are then filled in a PCR-like reaction employing Pfu DNA polymerase to generate blunt-ended molecules.
(0.03 MB DOC)

Table S2 Primers sets for PCR-based addition of sticky ends to zipper cDNAs.
(0.05 MB DOC)
