# Supplementary figures and images for: A Strategy for Adenovirus Vector Targeting with a Secreted Single Chain Antibody
Source: PLoS One. 2009 Dec 21;4(12):e8355. doi: 10.1371/journal.pone.0008355 (PMC2791226; doi:10.1371/journal.pone.0008355)

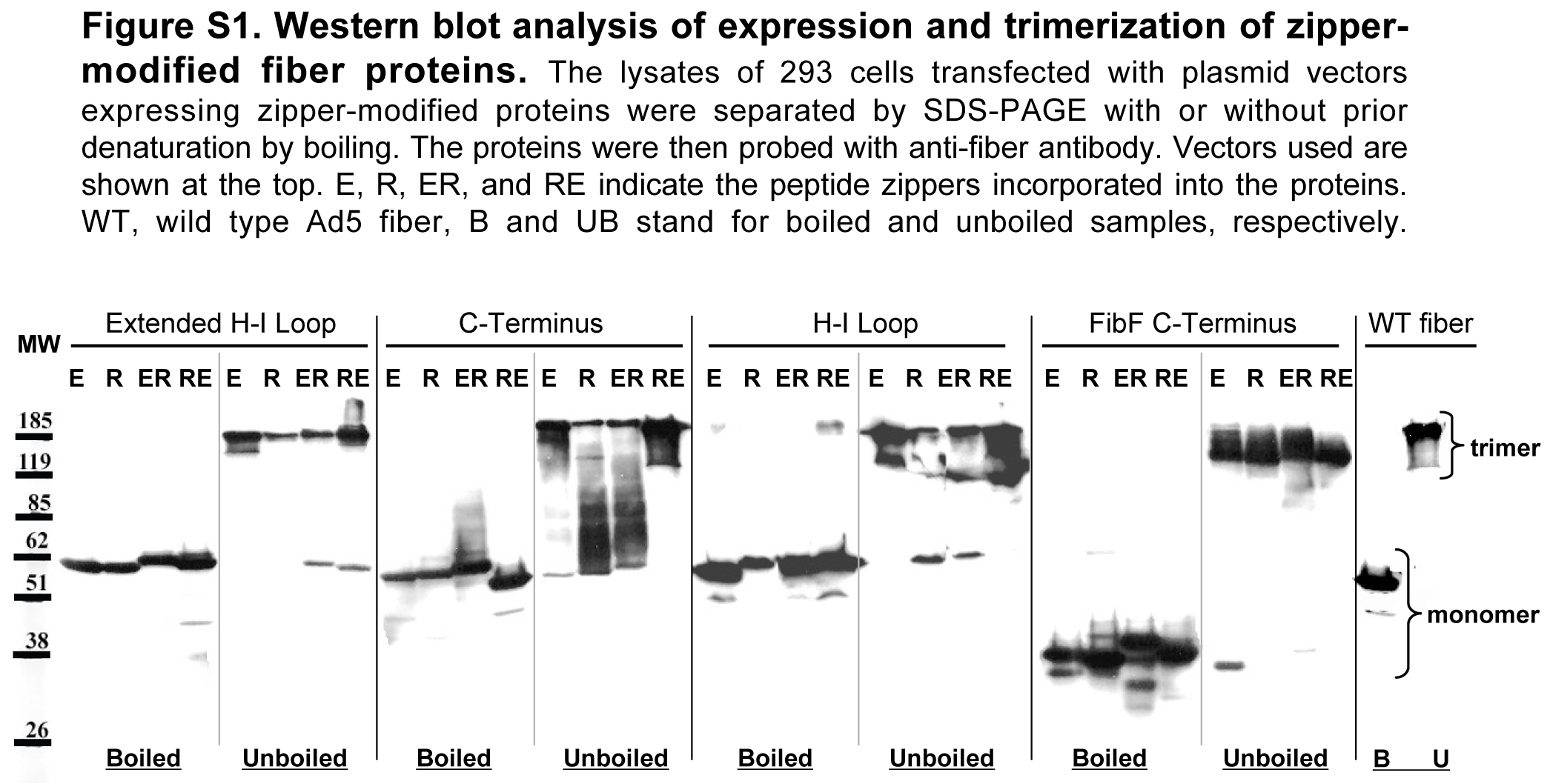

Supplement: Figure S1 — Western blot analysis of expression and trimerization of zipper-modified fiber proteins. The lysates of 293 cells transfected with plasmid vectors expressing zipper-modified proteins were separated by SDS-PAGE with or without prior denaturation by boiling. The proteins were then probed with anti-fiber antibody. Vectors used are shown at the top. E, R, ER, and RE indicate the peptide zippers incorporated into the proteins. WT, wild type Ad5 fiber, B and UB stand for boiled and unboiled samples, respectively. (0.42 MB TIF) [file pone.0008355.s001.tif]

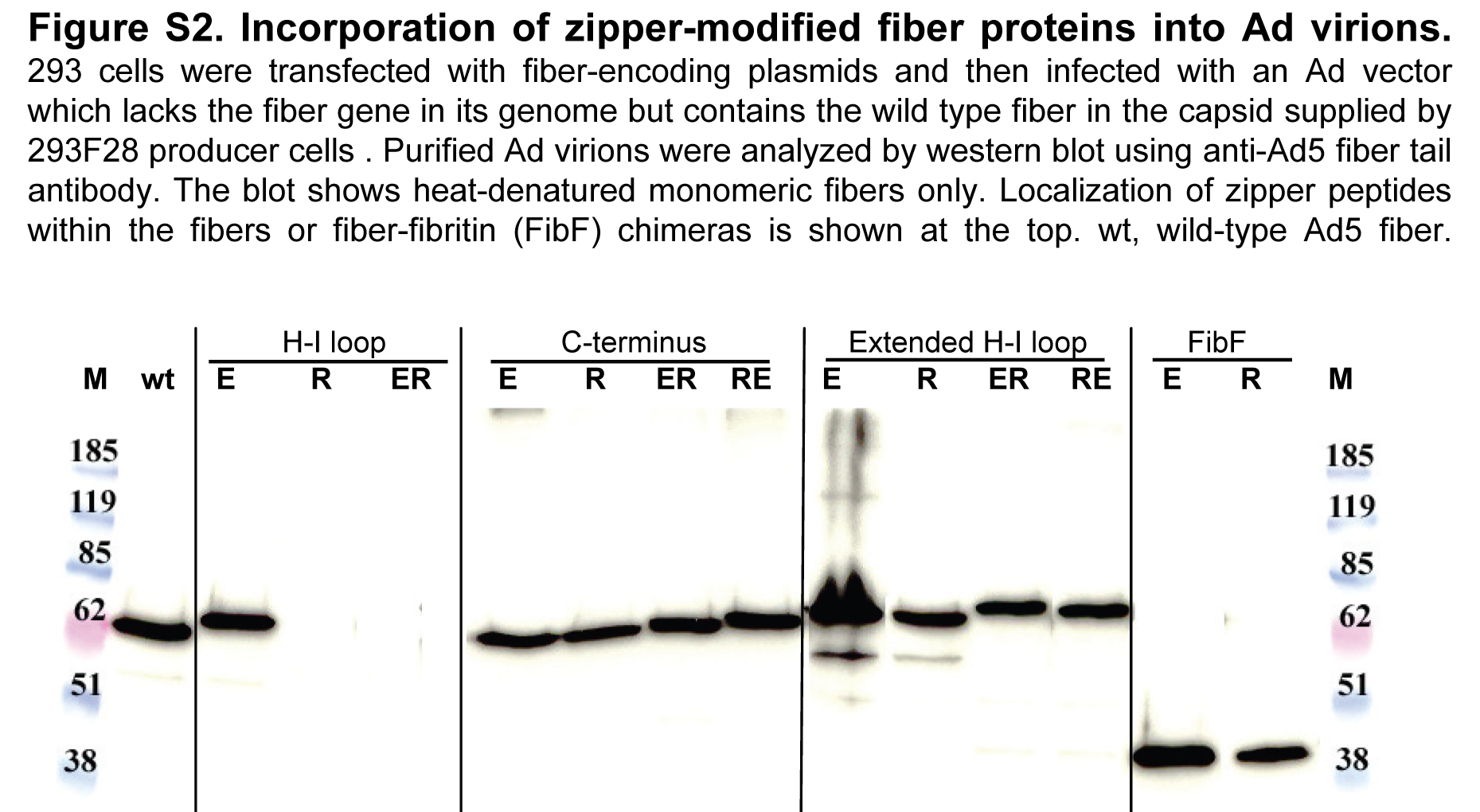

Supplement: Figure S2 — Incorporation of zipper-modified fiber proteins into Ad virions. 293 cells were transfected with fiber-encoding plasmids and then infected with an Ad vector which lacks the fiber gene in its genome but contains the wild type fiber in the capsid supplied by 293F28 producer cells. Purified Ad virions were analyzed by western blot using anti-Ad5 fiber tail antibody. The blot shows heat-denatured monomeric fibers only. Localization of zipper peptides within the fibers or fiber-fibritin (FibF) chimeras is shown at the top. wt, wild-type Ad5 fiber. (0.45 MB TIF) [file pone.0008355.s002.tif]

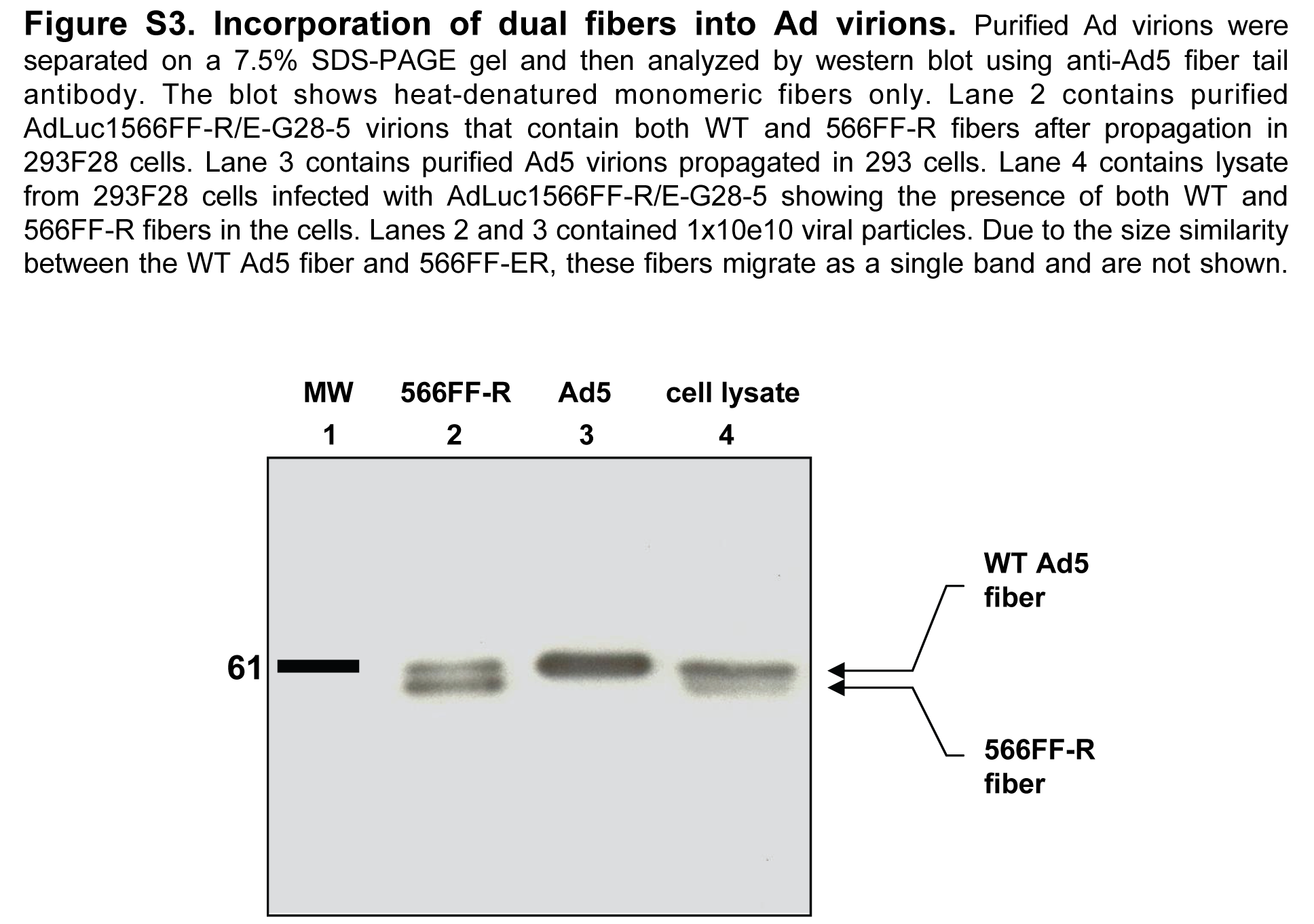

Supplement: Figure S3 — Incorporation of dual fibers into Ad virions. Purified Ad virions were separated on a 7.5% SDS-PAGE gel and then analyzed by western blot using anti-Ad5 fiber tail antibody. The blot shows heat-denatured monomeric fibers only. Lane 2 contains purified AdLuc1566FF-R/E-G28-5 virions that contain both WT and 566FF-R fibers after propagation in 293F28 cells. Lane 3 contains purified Ad5 virions propagated in 293 cells. Lane 4 contains lysate from 293F28 cells infected with AdLuc1566FF-R/E-G28-5 showing the presence of both WT and 566FF-R fibers in the cells. Lanes 2 and 3 contained 1x10e10 viral particles. Due to the size similarity between the WT Ad5 fiber and 566FF-ER, these fibers migrate as a single band and are not shown. (0.29 MB TIF) [file pone.0008355.s003.tif]
