# Supplementary material for: A Strategy for Adenovirus Vector Targeting with a Secreted Single Chain Antibody
Source: PLoS One. 2009 Dec 21;4(12):e8355. doi: 10.1371/journal.pone.0008355 (PMC2791226; doi:10.1371/journal.pone.0008355)
Supplement: Table S1 — Oligonucleotides used for assembling sequences encoding peptide zippers. When partially annealed, the underlined complementary nucleotides form a duplex within each zipper sequence. The recessed 3′-ends of the resultant duplexes are then filled in a PCR-like reaction employing Pfu DNA polymerase to generate blunt-ended molecules. (0.03 MB DOC) [file pone.0008355.s004.doc]

| Zipper | Oligonucleotides |
| --- | --- |
| E-E_34_ | 5’-CGTGCAGCTTTCCTGGAGAAGGAGAACACTGCACTGCGTACT |
|  | 5’-GATGTTCTCACATCGTCCTACCTCCTTCTCCAGTTCAGCTACCTCAGTACGCAGTG  CAGTGTTC |
| R-R_34_ | 5’-CGTGCAGCTTTCCTGGAGAAGGAGAACACTGCACTGCGTACT |
|  | 5’-GATGTTCCGACATCGTCCTACTCGCTTCCGCAGTTCAGCTACACGAGTACGCAGTG  CAGTGTTC |
| EE_12_RR_345_L | 5’-CTGGAGATCGAGGCAGCTTTCCTGGAACGGGAGAACACTGCACTGGAGACTCGTG  TAGCTGAACTGCGGCAGCGA |
|  | 5’-CAGAGGTCCGTAACGAGTTCGATACTGTGAGACTCGGTTCCGCAGACGCTGGACT  CGCTGCCGCAGTTCAGCTAC |
| RR_12_EE_345_L | 5’-CTGGAGATCCGTGCAGCTTTCCTGCGTCAACGGAACACTGCACTGCGTACTGAGG  TAGCTGAACTGGAGCAGGA |
|  | 5’-CAGAGGTCCGTAACGAGTTTCATACTGTGAGACTTCGTTCTCCAGACGCTGGACCT  CCTGCTCCAGTTCAGCTAC |

#### Table S1. Oligonucleotides used for assembling sequences encoding peptide zippers

When partially annealed, the underlined complementary nucleotides form a duplex within each zipper sequence. The recessed 3’-ends of the resultant duplexes are then filled in a PCR-like reaction employing *Pfu* DNA polymerase to generate blunt-ended molecules.
