# Supplementary material for: A Strategy for Adenovirus Vector Targeting with a Secreted Single Chain Antibody
Source: PLoS One. 2009 Dec 21;4(12):e8355. doi: 10.1371/journal.pone.0008355 (PMC2791226; doi:10.1371/journal.pone.0008355)
Supplement: Table S2 — Primers sets for PCR-based addition of sticky ends to zipper cDNAs. (0.05 MB DOC) [file pone.0008355.s005.doc]

#### Table S2. Primers sets for PCR-based addition of sticky ends to zipper cDNAs.

Sequences in **bold** comprise the sticky end nucleotides complementary to overhangs remaining following *Bae*I cleavage of pVS.ΔHI-BaeI. Non-bolded sequence is complementary to each zipper cDNA.

| E-E_34_ | F-long | **CAACT**CGTGCAGCTTTCCTGGAG |
| --- | --- | --- |
|  | R-long | **GATGG**GATGTTCTCACATCGTCC |
|  | F-short | CGTGCAGCTTTCCTGGAG |
|  | R-short | GATGTTCTCACATCGTCC |
| R-R_34_ | F-long | **CAACT**CGTGCAGCTTTCCTGGAG |
|  | R-long | **GATGG**GATGTTCCGACATCGTCC |
|  | F-short | CGTGCAGCTTTCCTGGAG |
|  | R-short | GATGTTCCGACATCGTCC |
| EE_12_RR_345_L | F-long | **CAACT**CTGGAGATCGAGGCAGCT |
|  | R-long | **GATGG**CAGAGGTCCGTAACGAGT |
|  | F-short | CTGGAGATCGAGGCAGCT |
|  | R-short | CAGAGGTCCGTAACGAGT |
| RR_12_EE_345_L | F-long | **CAACT**CTGGAGATCCGTGCAGCT |
|  | R-long | **GATGG**CAGAGGTCCGTAACGAGT |
|  | F-short | CTGGAGATCCGTGCAGCT |
|  | R-short | CAGAGGTCCGTAACGAGT |

Sequences in **bold** comprise the sticky end nucleotides complementary to overhangs remaining following *Bae*I cleavage of pVS-PB40_(BaeI)_. Non-bolded sequence is complementary to each zipper cDNA.

| E-E_34_ | F-long | **CCATT**CGTGCAGCTTTCCTGGAG |
| --- | --- | --- |
|  | R-long | **AAGGT**GATGTTCTCACATCGTCC |
|  | F-short | CGTGCAGCTTTCCTGGAG |
|  | R-short | GATGTTCTCACATCGTCC |
| R-R_34_ | F-long | **CCATT**CGTGCAGCTTTCCTGGAG |
|  | R-long | **AAGGT**GATGTTCCGACATCGTCC |
|  | F-short | CGTGCAGCTTTCCTGGAG |
|  | R-short | GATGTTCCGACATCGTCC |
| EE_12_RR_345_L | F-long | **CCATT**CTGGAGATCGAGGCAGCT |
|  | R-long | **AAGGT**CAGAGGTCCGTAACGAGT |
|  | F-short | CTGGAGATCGAGGCAGCT |
|  | R-short | CAGAGGTCCGTAACGAGT |
| RR_12_EE_345_L | F-long | **CCATT**CTGGAGATCCGTGCAGCT |
|  | R-long | **AAGGT**CAGAGGTCCGTAACGAGT |
|  | F-short | CTGGAGATCCGTGCAGCT |
|  | R-short | CAGAGGTCCGTAACGAGT |
